# The GIP gamma-tubulin complex-associated proteins are involved in nuclear architecture in *Arabidopsis thaliana*

**DOI:** 10.3389/fpls.2013.00480

**Published:** 2013-11-27

**Authors:** Morgane Batzenschlager, Kinda Masoud, Natacha Janski, Guy Houlné, Etienne Herzog, Jean-Luc Evrard, Nicolas Baumberger, Mathieu Erhardt, Yves Nominé, Bruno Kieffer, Anne-Catherine Schmit, Marie-Edith Chabouté

**Affiliations:** ^1^Centre National de la Recherche Scientifique, Institut de Biologie Moléculaire des Plantes, UPR 2357, Conventionné avec l'Université de StrasbourgStrasbourg, France; ^2^Biotechnologie et Signalisation cellulaire, Institut de Recherche de l'Ecole de Biotechnologie de Strasbourg, UMR 7242, Université de StrasbourgIllkirch, France; ^3^Institut de Génétique et Biologie Moléculaire et Cellulaire, Ecole Supérieure de Biotechnologie de StrasbourgIllkirch, France

**Keywords:** gamma-tubulin complex, AtGIP1/MOZART1, AtTSA1, nuclear envelope, *Arabidopsis thaliana*

## Abstract

During interphase, the microtubular cytoskeleton of cycling plant cells is organized in both cortical and perinuclear arrays. Perinuclear microtubules (MTs) are nucleated from γ-Tubulin Complexes (γ-TuCs) located at the surface of the nucleus. The molecular mechanisms of γ-TuC association to the nuclear envelope (NE) are currently unknown. The γ-TuC Protein 3 (GCP3)-Interacting Protein 1 (GIP1) is the smallest γ-TuC component identified so far. AtGIP1 and its homologous protein AtGIP2 participate in the localization of active γ-TuCs at interphasic and mitotic MT nucleation sites. Arabidopsis *gip1gip2* mutants are impaired in establishing a fully functional mitotic spindle and exhibit severe developmental defects. In this study, *gip1gip2* knock down mutants were further characterized at the cellular level. In addition to defects in both the localization of γ-TuC core proteins and MT fiber robustness, *gip1gip2* mutants exhibited a severe alteration of the nuclear shape associated with an abnormal distribution of the nuclear pore complexes. Simultaneously, they showed a misorganization of the inner nuclear membrane protein AtSUN1. Furthermore, AtGIP1 was identified as an interacting partner of AtTSA1 which was detected, like the AtGIP proteins, at the NE. These results provide the first evidence for the involvement of a γ-TuC component in both nuclear shaping and NE organization. Functional hypotheses are discussed in order to propose a model for a GIP-dependent nucleo-cytoplasmic continuum.

## Introduction

The nuclear envelope (NE) is a very specialized structure of eukaryotic cells separating the cytoplasm from the nucleoplasm. It consists of an outer and an inner nuclear membrane (INM) which join together at the sites where protein channels, corresponding to nuclear pore complexes (NPCs), mediate the traffic of macromolecules (Dultz and Ellenberg, 2007). In interphase cells, the ONM forms a continuous membrane system with the rough endoplasmic reticulum (ER), while the INM contains a subset of specific membrane-associated proteins (Dreger et al., 2001; Burke and Ellenberg, 2002). The NE regulates selective exchanges between the nucleoplasm and the cytosol but also provides anchoring sites for chromatin and the cytoskeleton (Hetzer et al., 2005). Indeed, recent data revealed a rather complex interplay between both compartments, not only through NPCs, but also through the establishment of a nucleo-cytoplasmic continuum, linking the cytoskeleton to the nucleoskeleton (Crisp et al., 2006; Ostlund et al., 2009). This continuum is based on the formation of specialized LINC (LInkers of Nucleoskeleton and Cytoskeleton) complexes. In various animal and fungi models, the core units of LINC complexes consist of KASH domain-containing proteins located in the outer nuclear membrane (ONM) and SUN domain proteins located in the INM, interacting with each other in the perinuclear space to form bridges across the NE. On the cytoplasmic side, the KASH domain proteins contact either directly actin filaments or indirectly microtubules (MTs) through motor proteins, such as dyneins and kinesins (Méjat and Misteli, 2010). On the nucleoplasmic side, SUN domain proteins interact with chromatin through lamins, lamin-binding proteins (except in plants and fungi) or specific INM associated proteins, thus organizing the nuclear content (Stuurman et al., 1998; Mekhail and Moazed, 2010).

In animals, interactions between SUN-KASH complexes and the cytoskeleton are largely involved in nuclear positioning, migration and anchorage (Crisp et al., 2006; Méjat and Misteli, 2010). They are also involved in tethering MT-Organizing Centers (MTOCs) to the NE. However, the molecular adaptors linking MTOCs to the ONM are poorly characterized so far. From the zygote stage to the early steps of *C. elegans* embryogenesis, the ZYG-12 KASH protein is recruited at the NE by SUN1/MTF-1, and mutations in the *ZYG-12* gene perturb the coupling between the nucleus and the centrosome (Malone et al., 2003). A connection between ZYG-12, dynein and MTs has been demonstrated, but remains hypothetical for the nuclear attachment of the centrosome/γ-TuCs (Malone et al., 2003; Zhou et al., 2009). In human fibroblasts, the emerin type II INM protein has also been found located at the ONM, where it plays a crucial role in the association of the centrosome to the outer NE through a MT-dependent mechanism. However, neither the nesprin-1 and nesprin-2 KASH domain proteins nor the SUN1 and SUN2 INM proteins seemed to be involved in this process (Salpingidou et al., 2007). To date, the role of SUN-KASH bridges linking the MTOC/centrosome to the NE remains unknown in mammals.

In plants, besides the NPC components (Tamura et al., 2010), the molecular characterization of NE proteins and their functional importance in a nucleo-cytoplasmic continuum have emerged. The first SUN proteins were characterized in rice, Arabidopsis and maize, respectively, (Moriguchi et al., 2005; Graumann et al., 2010; Murphy et al., 2010). More recently, the first plant KASH proteins were characterized in Arabidopsis and named WIPs (Zhao et al., 2008; Zhou et al., 2012). Arabidopsis WPP domain-interacting proteins (AtWIPs) are three tail-anchored ONM proteins which were shown to interact with Arabidopsis SUN1 and SUN2, thus defining the first LINC complex identified in plants (Zhou et al., 2012). AtWIPs redundantly anchor Arabidopsis RanGTPase-activating protein 1 (AtRanGAP1) to the outer NE (Xu et al., 2007), while mammalian RanGAP is anchored by the nucleoporin RanBP2 to control the trafficking of soluble proteins between the nucleoplasm and the cytoplasm (Mahajan et al., 1997). Interestingly, the lack of either AtSUN (Oda and Fukuda, 2011) or AtWIP proteins (Zhou et al., 2012) has only led to moderate phenotypes at the level of nuclear morphology in specific cell types, and no developmental or fertility defects have been observed. The first characterization of SUN-KASH interactions in plants indicates that, although LINC complexes are conserved among eukaryotes, they may have, at least partly, functionally diverged.

Until now, no relationship - neither with chromatin nor with γ-TuCs-has been established at the NE for a plant LINC complex. One of the functions of the NE is to act as an MTOC in the plant acentrosomal nucleation system (Stoppin et al., 1994; Canaday et al., 2000). Although γ-TuC components were shown to be recruited at the nuclear surface (Seltzer et al., 2007), their anchoring partners at the NE remain completely unknown. GIP proteins (AtGIP1 and AtGIP2) were discovered in Arabidopsis as partners of GCP3, one of the γ-TuC core subunits (Janski et al., 2008, 2012; Nakamura et al., 2012). AtGIPs localize to both cortical (Nakamura et al., 2012) and mitotic MT arrays and are required for proper γ-TuC localization, spindle integrity, and chromosomal segregation (Janski et al., 2012). The phenotype of *gip1gip2* knock down Arabidopsis mutants is severe and the complete KO is lethal (Nakamura et al., 2012), highlighting the functional importance of GIP proteins in Arabidopsis. These small proteins (around 7-8 kDa) are conserved among eukaryotes but have only been characterized in human (Hutchins et al., 2010) and more recently in *S. pombe* (Dhani et al., 2013; Masuda et al., 2013).

Here we present an exhaustive comparison of GIP proteins in unicellular and multicellular organisms and propose a structural model for Arabidopsis GIPs, partly validated by circular dichroism (CD). Beside their localization at MT mitotic arrays in dividing cells, AtGIPs are also present at the NE during interphase as a dotted pattern (Janski et al., 2012). At the subcellular level, we observed enlarged and deformed nuclei in the *gip1gip2* root tips. Such alterations of the nuclear shape were also observed in other tissues (in undifferentiated and differentiated cells) and were corroborated by a mislocalization of the AtSUN1 INM marker and a misdistribution of NPCs. A novel AtGIP1-interacting partner, AtTSA1, was identified through a yeast two-hybrid screen. Both AtGIP1 and AtTSA1 localized at the NE in Arabidopsis root tip cells. These results point out an important role played by AtGIP1, and possibly its associated proteins, in the nucleo-cytoplasmic interface organization and functions.

## Materials and methods

### Plant material and growth conditions

Arabidopsis transformation was performed using the floral dipping method (Clough and Bent, 1998) and the *Agrobacterium tumefaciens* strain GV3101 to produce GIP1::AtGIP1-GFP, GIP2::AtGIP2-GFP, 35S::AtSUN1-YFP, and 35S::AtTSA1-RFP transgenic lines. 35S::AtGIP1-GFP and 35S::AtGIP2-GFP Arabidopsis lines have been described previously (Janski et al., 2012). 35S::EYFP-AtCENH3 lines were described by Lermontova et al. (2006).

Wild-type and transgenic Arabidopsis lines were grown *in vitro* on Murashige and Skoog medium (SERVA Electrophoresis GmbH, Heidelberg, Germany) at 20°C in 12 h per day of 70 μmol m^−2^ s^−1^ fluorescent lighting, subcultured on soil and transferred to a growth chamber with light/dark cycles of 16h/8h for 2 weeks and then to the greenhouse.

### Construction of recombinant plasmids

Arabidopsis GIP1 and TSA1 cDNA and genomic fragments were generated by PCR using primers derived from the gene sequence (Table S1).

To express AtGIP1 in *Escherichia coli*, the AtGIP1 cDNA was cloned into the pET102D vector (*Invitrogen*™ Life Technologies, Saint Aubin, France). The sequences encoding thioredoxin and the 8 N-terminal AtGIP1 amino acids were deleted, and a sequence corresponding to a hexa-histine tag was introduced at the 5' of the AtGIP1 coding sequence, by PCR-based directed mutagenesis, to obtain pET(His)6AtGIP1.

The AtGIP1 cDNA was fused in-frame to the Gal4 DNA binding domain in the yeast vector pAS2ΔΔ (Institut Pasteur, Paris, France). Other yeast recombinant vectors, used in this study, have been described by Janski et al. (2008; 2012).

AtGIP1 or AtGIP2 promoter and coding sequences were cloned into pDONR™207 and transferred into the plant expression vector pGWB604 or pMDC107, respectively, by performing an LR reaction according to the manufacturer's protocol (Gateway® Technology Manual - *Invitrogen*™), to express GFP fusion proteins.

*AtTSA1* (*At1g52410.1*) cDNA in pDTSA1 (derived from pDONR™201-kindly provided by Pr. Takamasa Suzuki, Nagoya University, Japan) was introduced into the binary vector pH7RWG2 (Karimi et al., 2002), by performing an LR reaction according to the manufacturer's protocol (Gateway® Technology Manual-*Invitrogen*™), to express the AtTSA1-mRFP fusion. 35S::AtSUN1-YFP was cloned into pCAMBIA1300 as described previously by Zhou et al. (2012).

### Expression and purification of recombinant proteins

pET(His)6AtGIP1 was transformed into BL21(DE3) *E. coli* strain and expression was performed in an auto-inducing medium (Studier, 2005) at 20°C for 65 h. Cells were harvested by centrifugation and lysed in Tris 50 mM pH 8, NaCl 300 mM, glycerol 5%, urea 8 M, DTT 5 mM, Tween-20 0.1% by sonication. Lysate was clarified by centrifugation at 15000 *g* for 20 min and the protein purified by binding onto Ni-agarose resin (Protino NiNTA Macherey-Nagel, Hoerd, France). The purified protein was refolded by pulse dilution in Na phosphate buffer 10 mM pH 7.4, NaCl 25 mM, and 1 mg/ml of NVoy polymer (Expedeon, Harston, UK), followed by an incubation at 4°C for 16 h and 5 h at 25°C. Aggregated proteins were removed by centrifugation at 100 000 *g*, 1 h at 4°C. Refolded proteins were analyzed on SDS-PAGE gel and quantified by Bradford assay (Bio-Rad, Hercules, CA, USA) with BSA as standard.

### Circular dichroism

CD experiments were recorded on a Jasco J-815 spectropolarimeter (Easton, MD, USA) equipped with an automatic 6-position Peltier thermostated cell holder. The instrument was calibrated with 10-camphorsulphonic acid. Far-UV CD data were collected in the 182-260 nm range using a 0.1 mm pathlength cell (Quartz-Suprasil, Hellma UK Ltd, Southend on Sea, UK) at 25°C ± 0.1°C. Spectra were acquired using a continuous scan rate of 100 nm/min and were presented as an average of 25 successive scans. The response time and the bandwidth were 0.5 sec and 1 nm, respectively. The absorbances of the sample (at a concentration of 28 μM) and buffer were kept as low as possible. Spectra were obtained in 10 mM sodium phosphate buffer at pH 7.4 with 25 mM NaCl and 1mg/ml of Nvoy polymer. The spectrum was corrected by subtracting the solvent spectrum obtained under identical conditions. The secondary structure was estimated with the CDPro suite software (Sreerama and Woody, 2000). A far UV circular dichroism spectrum was used as described (Kelly et al., 2005). The spectrum of the AtGIP1 construct has been deposited in the Protein Circular Dichroism Data Bank (Whitmore et al., 2011) (http://pcddb.cryst.bbk.ac.uk) with the accession code CD0004243000 for release upon publication.

### Yeast two-hybrid assays

The CG1945 haploid Mat a yeast strain was transformed with the pAS2ΔΔ-AtGIP1 recombinant plasmid. The screening of an *A. thaliana* cDNA library (Clontech, Saint-Germain-en-Laye, France) was carried out as described by Janski et al. (2008). Candidate yeast colonies were selected on a minimal medium lacking histidine, tryptophan and leucine, and supplemented with 20 mM of 3-amino-1, 2, 4-triazole (3-AT). Prey plasmids were isolated from positive colonies, reintroduced into the AH109 yeast strain and re-assayed for growth on selective media, using various stringency, according to the Clontech Matchmaker user manual.

### TEM and immunolocalization

Root tissue samples were taken from 9-day old Arabidopsis seedlings, fixed overnight in 1.5% glutaraldehyde, 2 h post-fixed with 0.1% (v/v) osmium tetroxyde in 150 mM phosphate buffer and stained overnight with 2% uranyl acetate (modified Seltzer et al., 2007). Samples were dehydrated through an ethanol series, then infiltrated with EPON 812 medium grade resin (Polysciences Inc, Eppelheim, Germany) and polymerized for 48 h at 60°C. Ultrathin sections (70 nm) were cut using an ultracut E microtome (Reichert, Wien, Austria) and collected on grids coated with formvar (Electron Microscopy Science, Fort Washington, PA, USA). Immunolocalization of AtGIP-GFP fusion proteins was performed by incubating the sections with a rabbit anti-GFP antibody (A11122, *Invitrogen*™) diluted 1/500 in 1% bovine serum albumin in phosphate buffer saline (PBS) for 2 h at room temperature. After repeated washing with PBS, the sections were then incubated for 2 h with goat-anti-rabbit antibodies coupled to 15 nm colloidal gold particles (Aurion EM Reagents, Wageningen, The Netherlands) followed by washing with PBS and H_2_O. Samples were visualized with a Hitachi H-600 electron microscope operating at 75 kV and Images were captured with a CCD Advantage HR Hamamatsu camera and AMT software (Advanced Microscopy Techniques, Danvers, MA, USA).

### Immunolocalization on root tip cells

Arabidopsis seedlings were fixed for 40min in 1.5% paraformaldehyde and 0.5% glutaraldehyde in PEMT buffer (50 mM PIPES, 2 mM EGTA, 2 mM MgSO4, 0.05% Triton X-100, pH 7.2), and then treated as described by Erhardt et al. (2002). Unspecific binding was blocked by incubation in PBS containing 2% Bovine serum albumin and 0.1% Triton X-100 (IF buffer) for 10 min. Primary and secondary antibodies were diluted in IF buffer and incubated for 1h at room temperature. The primary antibodies used in this study were the rabbit polyclonal anti-AtGIP1 (1/1000) (Janski et al., 2012), monoclonal anti-α-tubulin (clone DM1A, Sigma-Aldrich, St Louis, MO, USA) (1/6000), and polyclonal anti-AtSUN1 (1/1000) (kindly provided by D. Evans). Alexa 488- and Alexa 568-conjugated goat anti-rabbit IgG and goat anti-mouse IgG secondary antibodies (1:300) were purchased from Molecular Probes® (Life Technologies, Saint Aubin, France). DNA was stained using 0.1 μg/mL 4′, 6-diamidino-2-phenylindole dihydrochloride (DAPI).

### Fluorescence microscopy

The fluorescence of AtGIP1-GFP, AtSUN1-YFP, and AtTSA1-RFP was visualized on living seedlings mounted in water. Seedlings and immunostained cells were observed with a Zeiss LSM 780 confocal microscope in multitracking mode which is able to specifically discriminate each fluorochrome signature (Carl Zeiss AG, Le Pecq, France).

### Accession numbers

**Figure 1:**
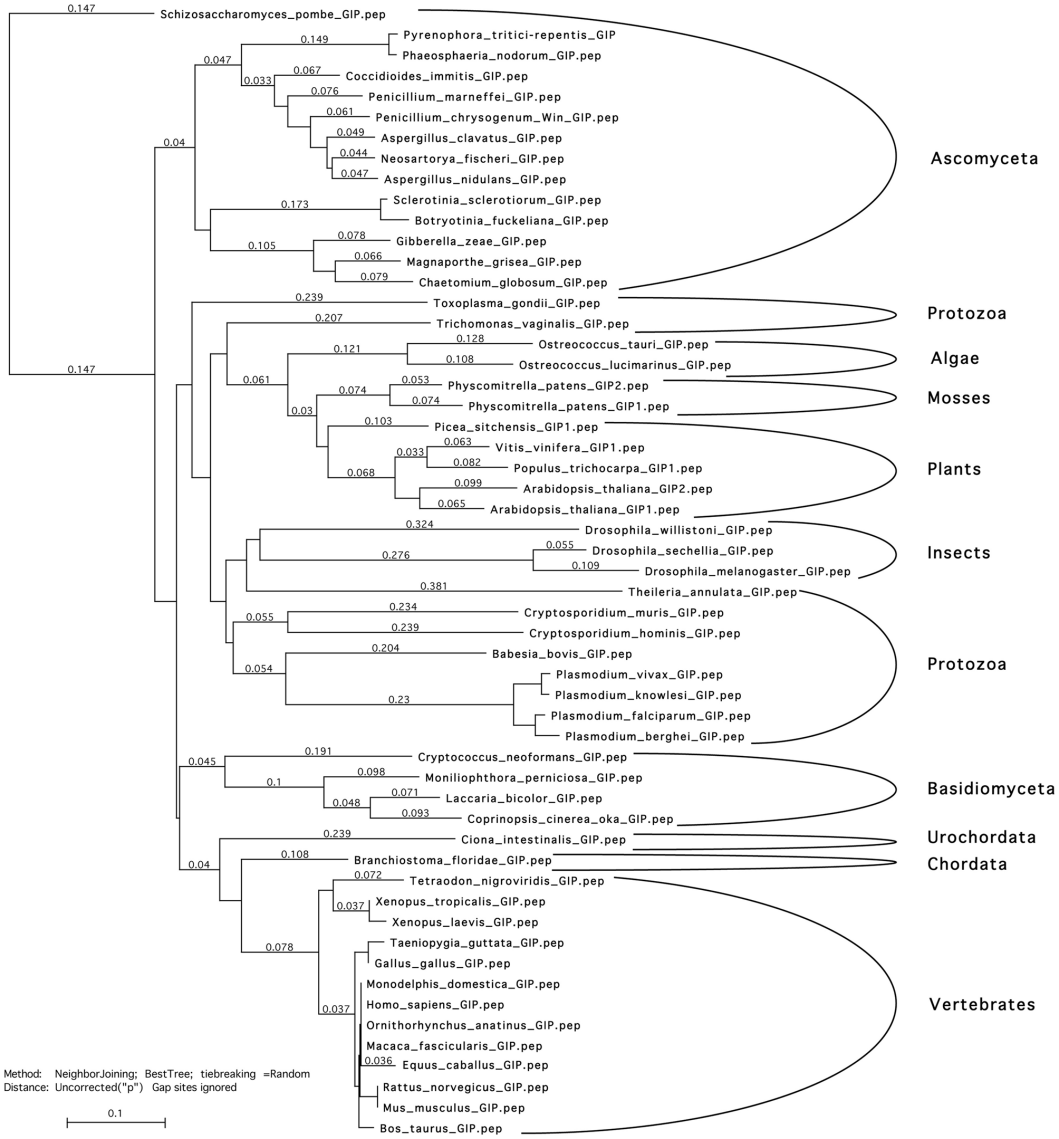
**Phylogenetic tree of GIP peptidic sequences**. Sequences were aligned using *T*-coffee and Myers-Miller matrix. A tree was constructed using the neighbor joining algorithm. Analyses were performed using the MacVector software. GIP sequences segregate accordingly to the genus of life evolution. This suggests that GIPs probably originate from one and the same ancestor and that they are not the products of a convergent evolution that would result in a mix of the sequences. Accessions numbers of the used sequences are presented in Table S2.

Sequence data from this study can be found in the Arabidopsis Genome Initiative or GenBank/EMBL databases under the following accession numbers: Arabidopsis (At) GIP1: At4g09550; AtGIP2: At1g73790; AtTSA1: At1g52410; AtNAI2: At3g15950; At3g15960; AtSUN1: At5g04990. Accession numbers of the sequences used for establishing the phylogenetic tree of GIP peptidic sequences (Figure 1) are presented in Table S2. CD data deposit on the accession number CD0004243000.

## Results

### AtGIPs are α-helix proteins conserved in unicellular and multicellular organisms

**Figure 2:**
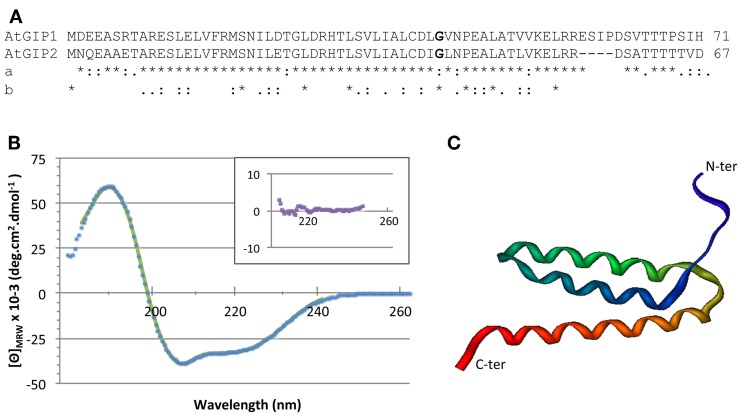
**GIP sequences and three dimensional structure. (A)** Arabidopsis GIP1 and GIP2 sequence alignment. Comparison **(a)** between both Arabidopsis sequences and **(b)** between AtGIP1 and other eukaryotes. (*) identical aminoacids, (:) similar aa, (.) semi-conserved aa. The conserved glycine residue (aa 42) is shown in bold print. **(B)** The structural integrity of AtGIP1 protein was analyzed using circular dichroism spectroscopy. Spectra were recorded at 25° C using recombinant AtGIP1 solution at 28 μM in 10 mM sodium phosphate buffer (pH 7.4), complemented with 25 mM NaCl and 1 mg/ml of Nvoy polymer. The signal is expressed in mean residue ellipticity (deg.cm^2^.dmol^−1^). Experimental data and fitting results are shown in dots and solid line, respectively. Inset shows the residuals using an expanded y-axis to better display the random distribution. **(C)** An AtGIP protein model generated with the LOMETS online server (http://zhanglab.ccmb.med.umich.edu/LOMETS/) and the methods of LOMETS and MODELLER v9.3 (Wu and Zhang, 2007) in a fully automated procedure. The predicted AtGIP1 3D structure is composed of 3 α-helices (aa 9–20; aa 25–40; aa 45–61).

AtGIPs were previously identified as partners of AtGCP3, a member of the γ-TuC (Janski et al., 2008). These small proteins have been identified in numerous organisms, as shown in Figure 1 and Table S2. The following sequence-[MILVT] xxx[LV] xxx [LIVT]- [DTS] xxx [LIFVM]xx[CLIAVF]-[ILVME]-x [LMFVA]xxxGxx [PGA], xx [LIV]-[VIAS]x [VILAM]-[VIFLG]-has been established as a GIP signature. Used in database screens, it allowed us to identify GIPs in a large variety of eukaryote species, except *S. cerevisae*. In the plant kingdom, a GIP gene duplication occurrence was linked to the conquest of terrestrial habitats. AtGIP1, used as a reference, shares 41 to 78% identity with the 115 plant GIP sequences analyzed so far. Aminoacid similarities were mapped between AtGIP1 and AtGIP2 or human GIP (Figure 2A). GIPs do not harbor any characterized consensus functional domains, except a glycine (Figure 2A, in bold) and repeated leucines, mostly conserved throughout evolution (Figure 2A, stars).

In order to determine the AtGIP1 secondary structure content, we recorded a far UV circular dichroism spectrum. The secondary structure was estimated with the CDPro suite software using CD data of the SP37 reference database (a base of 37 soluble proteins of known secondary structure elements) and the CONTIN method. CONTIN fits the CD of the GIP protein by comparison with a linear combination of the spectra from a large database of proteins with known conformations. This led to 82% for α-helices, 4% for β-sheets and 14% for other secondary structure contents. These results indicate that α-helices mainly constitute AtGIP1 (Figure 2B). Accordingly, computer modeling predicts that the AtGIP1 3D structure is composed of three α-helices (Figure 2C). Interestingly, the conserved glycine residue (aa 42 in AtGIPs), found in all organisms except *S. pombe*, may constitute a glycine elbow needed for flexibility in the relative positioning of the three α-helices within the predicted 3D structure (Figure 2C).

These sequence analyses emphasize the wide conservation of GIP proteins from fungi to humans, with the presence of a specific sequence signature, which very likely reveals their biological relevance in eukaryotic cells.

### Functions of AtGIPs at the NE

Previous localization studies have shown that AtGIP1-GFP is present in the cytoplasm, the nucleoplasm and at the NE in interphase cells (Janski et al., 2012).

**Figure 3:**
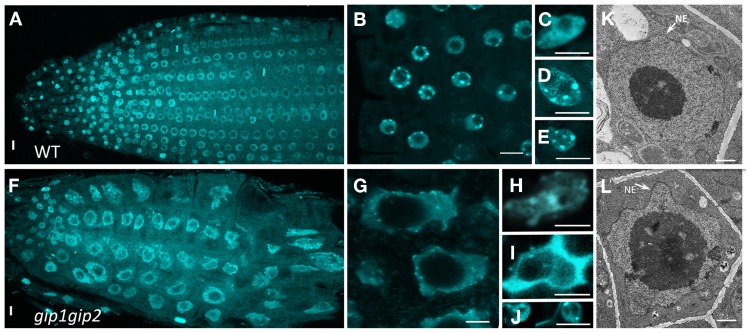
**Nuclear shape and DNA labeling in WT (A–E) and *gip1gip2* mutants (F–J) using DAPI staining. (A,F)** General view of a root tip. **(B,G)** Enlarged view of root tip meristematic cells. **(C,H)** Cotyledon nuclei. **(D,I)** Leaf nuclei. **(E,J)** Petal nuclei. The nuclei of *gip1gip2* mutant cells exhibit an increased size and are highly deformed. Bars = 5 μm. **(K,L)** TEM performed on WT and *gip1gip2* mutant root tip seedlings, respectively, showing NE deformations in the mutant **(L,** arrow**)**. Bars = 500 nm.

**Figure 4:**
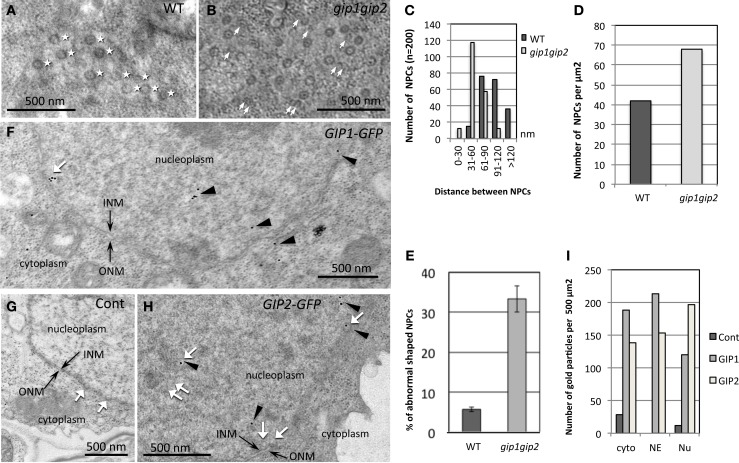
**Modification of NPC distribution in *gip1gip2* mutants and AtGIP distribution in cells expressing AtGIP-GFP constructs. (A,B)** Tangential views of the nuclear surface in TEM with NPC repartition (white stars) which is regular in WT **(A)**. Heterogenous distribution of NPCs and abnormal shaped NPCs (white arrows) in *gip1gip2* mutants **(B)**. **(C)** Analysis of the distances between NPCs in WT and mutants (*p*-value < 0.001). **(D)** Quantification of NPC number per μm^2^ in WT and mutants. **(E)** Quantification of abnormal NPCs. **(F–H)** Immunolabeling using anti-GFP antibodies (arrowheads) on cells expressing AtGIP1-GFP **(F)** or AtGIP2-GFP **(H)** and WT control cells **(G)**. **(I)** The quantification of gold particles was performed on 50 images corresponding to 3 independent experiments. Control cells which do not express a GFP fusion protein were labeled in parallel. Counting was performed in different subcellular compartments (Cytoplasm: cyto, NE and Nucleoplasm: Nu). Bars = 500 nm.

To further investigate the role of AtGIPs at the NE, morphological modifications of the nucleus were analyzed in the *gip1gip2* Arabidopsis mutants. Our observations revealed a drastic discrepancy in the size and shape of the mutant nuclei compared to WT (Figures 3A,F). Differences in size are likely due to the increase of ploidy previously described by FACS analyses (Janski et al., 2012). The difference in shape corresponds to a dramatic shift from a roundish shape to lobulated and highly dented nuclei. Using transmission electron microscopy (TEM) analyses, the NE of *gip1gip2* nuclei was shown to be deeply invaginated with protrusion compared to WT (Figures 3K,L). This phenotype was not limited to dividing cells (meristematic root cells, young leaves) but was also observed in various differentiated cells (mature cotyledon, petal), (Figures 3C–E, H–J). The quantification of these abnormalities was determined in root tips, where more than 70 % of the nuclei showed irregular shaping (Figure 3G). As NE defects may also affect NPC density, we investigated the NPC distribution using TEM in WT Arabidopsis and *gip1gip2* mutant root tips. In the NE of WT nuclei, the NPCs were evenly distributed and circular (Figure 4A). In comparison to WT nuclei in which the mean distance between two adjacent NPCs is of about 90 nm, this distance drops below 60 nm in the mutants. In about 33% of *gip1gip2* nuclei (*n* = 400 *p*-value < 0.01) the NPC shape was altered (Figures 4B,E). In mutants, 17% of NPCs were separated by 0 to 30 nm only (Figure 4C) and some of them were fused together, indicating spacing abnormalities (white arrows in Figure 4B). The number of NPCs was evaluated in an total area of 2 μm^2^ (Figure 4D). It shows an increase of 1.75 times in the mutant. This raises the question of AtGIP location at the NPC. We therefore investigated the localization of AtGIP1-GFP and AtGIP2-GFP in transgenic Arabidopsis lines, using TEM and immunogold labeling using anti-GFP antibodies. Both fusion proteins were found at the NE, within and /or closely associated with the NPC (17% and 7% of gold particles for AtGIP1 and AtGIP2, respectively), and were also located in the nucleoplasm and the cytoplasm (Figures 4F,H, arrowheads and I). As expected, very few aspecific signals were observed in control cells (Figures 4G,I). These results confirm our previous observation of GIP-GFP distribution in fluorescence (Janski et al., 2012).

**Figure 5:**
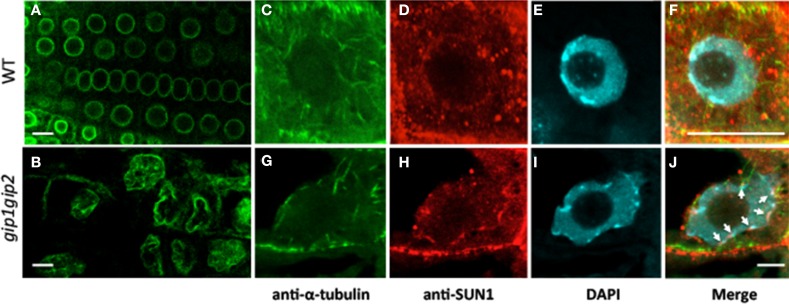
**Modification of the NE shape and organization**. AtSUN1-YFP reveals the INM when expressed in WT Arabidopsis **(A)** and *gip1gip2* mutants **(B)**. Note the altered nuclear shape with lobulations, and mislocalization of AtSUN1 in the nucleoplasm of mutant cells. Immunostaining of endogenous AtSUN1 **(D,H)** compared with MTs **(C,G)** and DAPI-stained DNA **(E,I)** in WT and *gip1gip2* root nuclei, respectively. Corresponding merged pictures **(F,J)** show a disrupted perinuclear localization of AtSUN1 in *gip1gip2* mutants **(J**, arrows**)** compared to WT **(F)**. Bars = 10 μm.

Alterations of the NE may be correlated to nuclear membrane protein reorganization. We have investigated whether the INM marker AtSUN1 was distributed differently in *gip1gip2* mutants and in WT nuclei. To this end, we introgressed the 35S::AtSUN1-YFP construct into a *gip1gip2* background. As expected, AtSUN1-YFP labeling first confirmed dramatic abnormally shaped nuclei in the mutants, compared to those observed in WT (Figures 5A,B). It secondly revealed an uneven AtSUN1 distribution through variability of fluorescence density at the membrane. Using an immunocytochemical approach with an antibody directed against AtSUN1, a similar phenotype on root tip nuclei was revealed in *gip1gip2* mutants, where AtSUN1 clusters appeared accumulated irregularly at the NE (Figure 5G, arrows). In WT cells, on the contrary, AtSUN1 was distributed uniformly at the INM, often with highly fluorescent dots where MTs emerged (Figures 5C–E).

AtGIP1-GFP localization as a spotty pattern at the NE (Janski et al., 2012) may be correlated to the MT nucleation sites dispersed at the nuclear surface. To further investigate the localization of AtGIP at the nucleo-cytoplasmic interface, the root tips of AtGIP1-GFP Arabidopsis seedlings were immunolabeled with anti-α-tubulin antibodies and incubated with DAPI. The observations of interphase cells revealed an AtGIP1-GFP dotted localization at the minus ends of some perinuclear MTs (Figure S1). Such dotted structures were, in certain cases, located close to bright DAPI stained heterochromatin. Interestingly, in root tip cells of an Arabidopsis line expressing the CENH3 centromeric histone fused to EYFP, some MT minus ends were also close to the EYFP-AtCENH3 signals observed at the nuclear periphery on chromocenters.

Our observations converge to a significant role of AtGIPs in both NE shaping and architecture, in addition to their function as γ-TuC components.

### AtGIP1 interacts with AtTSA1, a NE and ER-located protein

**Figure 6:**
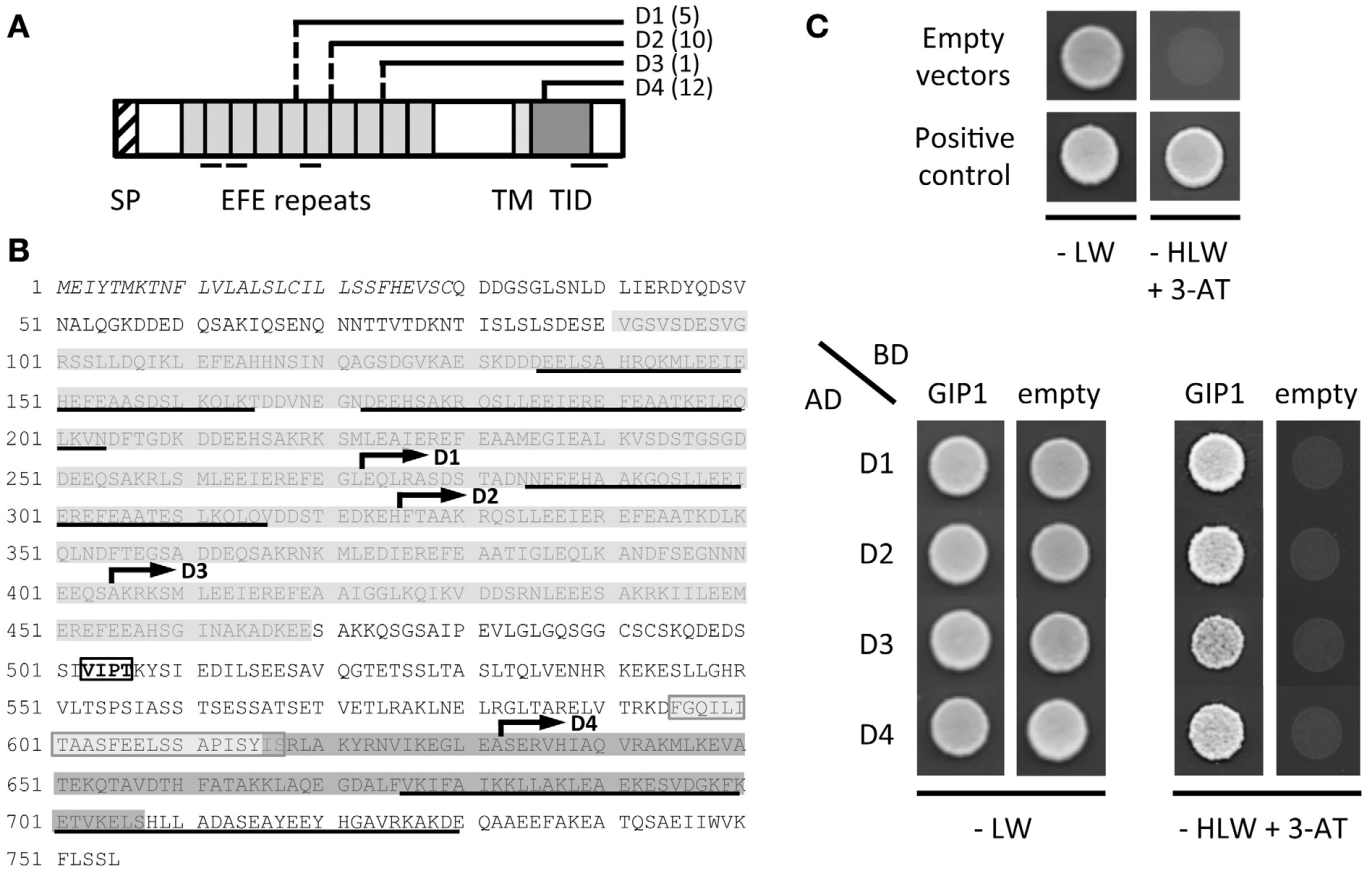
**AtGIP1 interacts with C-terminal domains of AtTSA1 in a yeast two-hybrid system. (A)** Schematic representation of AtTSA1 protein and corresponding C-terminal domains identified by yeast two-hybrid screening. The features of AtTSA1 protein have been initially described in Suzuki et al. (2005). SP, signal peptide; EFE repeats, multimerization and calcium-binding repeat sequence; TM, putative transmembrane domain; TID, TSK-interacting domain. Several coiled-coil motifs predicted by the Paircoil2 software (McDonnell et al., 2006) are also underlined. D1 to D4 regions correspond to the C-terminal portion of AtTSA1 with varying lengths toward the N-terminus (depicted by solid and dashed lines). Corresponding cDNAs have been repeatedly identified by yeast two-hybrid screening using AtGIP1 as a bait (the number of positive clones identified for each domain is indicated in brackets). **(B)** AtTSA1 amino acid sequence. Features of the protein represented in **(A)** are reported on AtTSA1 primary sequence as follows: SP (italics), EFE repeats (gray), TM (light gray), TID (dark gray), coiled-coil motifs (underlined). D1 to D4 domains are indicated by an arrow starting from the first corresponding amino acid (D1: 273 to 755; D2: 326 to 755; D3: 405 to 755; D4: 633 to 755). VIPT motif (boxed text): putative NE localization signal (Zhou et al., 2012; see text). **(C)** Interaction of AtGIP1 with AtTSA1 domains in a yeast two-hybrid assay. AH109 cells co-transformed with bait (Gal4 Binding Domain, BD) and prey (Gal4 Activation Domain, AD) recombinant plasmids were spotted directly on control (−LW) and selection (−HLW + 3-AminoTriazole, 3-AT) plates and grown for 2 days at 30°C. Upper panel: negative (empty vectors) and positive (pBWRepA/pGADRb1; Xie et al., 1996) interaction controls. Lower panel: interaction test. Transformants coding for D1 to D4 C-terminal regions of AtTSA1 grow in the presence of AtGIP1 on selective medium, indicating an interaction between the different proteins.

The full-length AtGIP1 protein was used as a bait to screen a three-week-old Arabidopsis cDNA library in a yeast two-hybrid approach. Twenty-eight clones, positive for β-galactosidase activity, contained the coding sequence of *TonSoKu (TSK)-associating protein 1* (*TSA1*)/*At1g52410* gene with varying lengths toward the 5'-terminus. The *AtTSA1* (*At1g52410.1)* gene encodes a 755-amino acid protein (Figures 6A,B). Two homologous genes were found in Arabidopsis, NAI2 *(At3g15950)* and the At3g15960 protein (Figure S2). Yeast co-transformation was performed with rescued library plasmids to confirm the interaction between AtGIP1 and AtTSA1 under nutritional selection. The growth of yeast cells harboring the positive clones, coding for various AtTSA1 C-terminal domains (D1 to D4, Figures 6A,B), is shown in Figure 6C.

Yeast cells expressing the D1 to D4 C-terminal regions of AtTSA1 grew efficiently in the presence of AtGIP1 under nutritional selection, confirming the interaction between the two proteins in a yeast two-hybrid system (Figure 6C). The minimal domain of AtTSA1 interacting with AtGIP1 corresponds to the C-terminal part (123 last aminoacids) of the protein (D4, Figures 6A,B). This domain partially overlaps the AtTSA1 domain interacting with AtTSK (aa 616-707; Suzuki et al., 2005). The D4 minimal AtTSA1 domain was also shown to strongly interact with AtGIP2 in a yeast growth assay carried out on selective media of increasing stringency (Figure S3).

**Figure 7:**
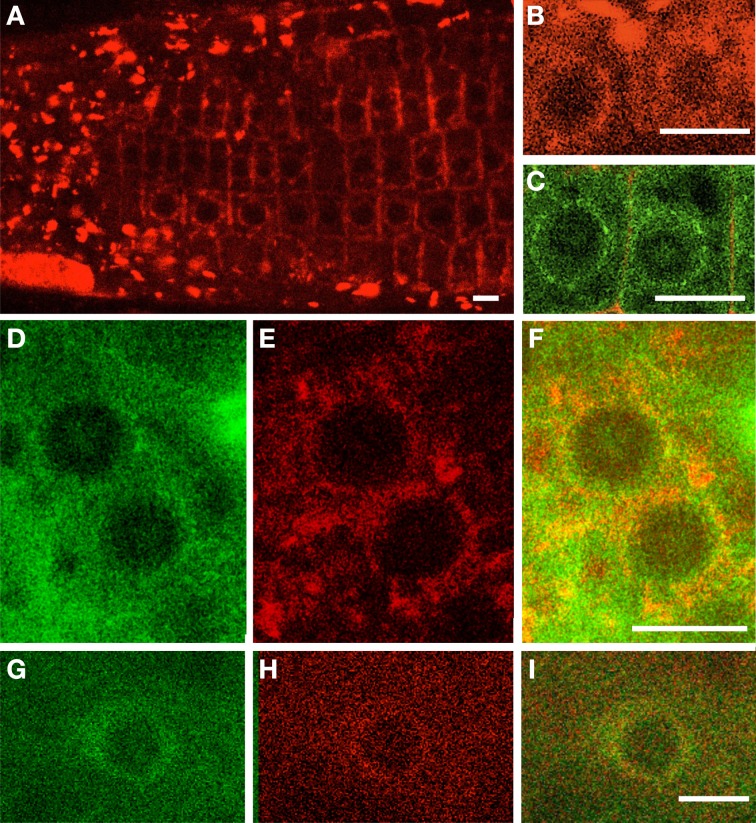
**Distribution of AtGIP and AtTSA1 in Arabidopsis root cells**. 35S::AtTSA1-RFP **(A,B)** and 35S::AtGIP1-GFP **(C)** constructs were expressed independently or simultaneously **(D–I)** in Arabidopsis rootlets. Individual pictures of AtGIP1-GFP **(D,G)**, AtTSA1-RFP **(E,H)** localization are merged **(F,I)**. AtTSA1 localizes in ER body-like structures and at the nuclear periphery **(A,B,E,H)**. AtGIPs and AtTSA1 proteins localize at the NE. Colocalization is observed on the merged images **(F,I)**. Bars = 10 μm.

In order to study the relationship between AtGIP1 and AtTSA1 *in vivo*, the subcellular localization of AtTSA1 was investigated using stable Arabidopsis transgenic lines expressing the 35S::AtTSA1-RFP construct. In root interphase cells displaying a moderate fluorescence signal, AtTSA1-RFP was found at the NE and in the form of ER body-like structures (Figures 7A,B,E,H). Interestingly, AtTSA1-RFP showed a similar pattern of NE localization compared to that of AtGIP1-GFP (Figures 7C,D,G). We then introgressed the 35S::AtTSA1-RFP construct in AtGIP1-GFP lines. Such transgenic plants expressing AtTSA1-RFP and AtGIP1-GFP displayed a colocalization of both proteins at specific areas of the NE in interphase root cells. (Figures 7F,I).

In conclusion, AtTSA1 was identified as a possible AtGIP-interacting partner at the NE, suggesting the participation of both proteins in structural links between the NE and the cytoskeleton.

## Discussion

### AtGIP structure and functional modeling

Analyses using CD (Figure 2) indicate that AtGIP1 is mainly formed by α-helices. Furthermore, computer modeling predicts that AtGIP1 may adopt a 3D structure composed of three central α-helices, leaving the N- and C-terminal domains less structured. Such a conformation could be assumed by other eukaryotic GIPs, considering the high degree of conservation of their central domain. In addition, a conserved putative glycine elbow in AtGIP1 may be of particular importance in modulating the 3D positioning of α-helices and in establishing interactions with partners such as AtGCP3, AtTSA1, and/or AtGIPs themselves. It must be noted that, AtGIP1-AtGIP1 and AtGIP1-AtGIP2 interactions were detected using a sensitive β-galactosidase lift assay, indicating possible homo- and heterotypic interactions between AtGIP proteins (Figure S4). AtGIP1 multimers were also detected during recombinant protein purification (data not shown), data which are in agreement with the oligomeric species described for MZT1/Tam4, the GIP1 homolog of *S. pombe* (Dhani et al., 2013). In fission yeast, it was speculated that the oligomerization of MZT1 might regulate the γ-TuC activity. Interestingly, AtGIPs (Janski et al., 2012) and MZT1/Tam4 (Dhani et al., 2013) interact with the N-terminal region of GCP3, arguing in favor of a role of GIPs as modulators of GCP3 activity. Due to their small size and multimerization propensity, GIPs could act as efficient adaptors of γ-TuCs.

### Function of microtubular/MTOC-associated AtGIP1

AtGIPs exhibit a punctate localization at the NE (Figure 7), resembling that of AtGCP3, a core component of the γ-TuC (Seltzer et al., 2007). NE localization of GIP/MZT1/Tam4 was also observed in *S. pombe*, with remnant MTs often found at the outer nuclear periphery (Masuda et al., 2013). This suggests that GIPs may be recruited at the NE as part of γ-TuCs. As fewer γ-TuC components (GCP4, GCP3 and γ-tubulin) were recruited to MT nucleation sites (nuclear surface, spindle) in *gip1gip2* mutants (Janski et al., 2012), AtGIPs may be crucial for anchoring γ-TuCs at MTOC sites, as was recently suggested in *S. pombe* (Masuda et al., 2013). The observations of AtGIP1-GFP at the minus end of perinuclear MTs reinforce such a hypothesis (Figure S1). In addition, AtGIP1 localization was close to NE underlying chromocenters. In Arabidopsis, chromocenters mainly consist of heterochromatin, previously described as associated with centromeres (Fransz et al., 2002; Soppe et al., 2002). Such observations suggest a possible mechanism in which a redistribution of MT forces could be involved in centromere mobility in the nucleoplasm, until they reach the nuclear periphery as observed in Figure S1. This would anticipate the further access of kinetochores/centromeres to MTs after NE breakdown, allowing rapid spindle building. Indeed, in higher plant prophase cells, pro-spindle MTs exert pushing forces which often leads to MT-loaded polar NE invaginations (Bajer and Mole-Bajer, 1969; Dixit and Cyr, 2002). These MTs often directly catch kinetochores at prometaphase onset, building kinetochore fibers. The intranuclear centromere mobility may therefore be linked to MT reorganization during G2 phase through the establishment of a GIP-dependent nucleo-cytoplasmic continuum.

### Involvement of AtGIPs in nuclear shaping and organization

Our understanding of the mechanisms determining the nuclear shape and size is still unclear. Intriguingly, *gip1gip2* mutant cells showed abnormal shaped nuclei (Figure 3) as observed in human diseases, such as envelopathies and cancers (Worman et al., 2010; Starr, 2012), In plants, the nuclear morphology varies from circular or slightly oval to spindle shaped, according to cell type and tissue specificities. Recently, the myosin XI-i motor protein was identified as a molecular adaptor between the actin cytoskeleton and the WIT-WIP-SUN bridge in Arabidopsis (Tamura et al., 2013). Contrary to the dramatic nuclear phenotypes observed in all tissues of *gip1gip2* mutants, *sun1, wip*, and *myosin XI-i* mutants led to only mild changes in the nuclear morphology of differentiated tissues, switching from the elongated shape to more roundish nuclei (Zhou et al., 2012; Tamura et al., 2013). These data suggest that AtGIPs are involved in specific complexes which may connect MT nucleation complexes to integral NE components. However, as the altered nuclear phenotypes cannot exclusively be explained by disrupted MT connections, plant GIPs may be involved in other functions, directly or indirectly linked to the NE environment. Indeed, the chromocenter size was disturbed in the *gip1gip2* mutant (see Figure 3) and GIPs were also found in the nucleoplasm close to heterochromatin (Figure 4).

Therefore, despite a role of AtGIPs at the ONM for helping γ-TuC anchoring, we cannot exclude a role of AtGIPs at the INM after diffusion through the nuclear pores, considering their small size. At the INM of human nuclei, SUN proteins connect chromatin through interactions with lamins and/or lamin-associated proteins of the lamina (Méjat and Misteli, 2010). In plants, despite the existence of a lamina-like structure (plamina) with NMCP/LINC components described as lamin-like proteins (Dittmer et al., 2007; Fiserova et al., 2009; Moreno Diaz de la Espina, 2009; Ciska and Moreno Diaz de la Espina, 2013), the interaction of NMCP/LINC with chromatin has not been established so far. Even though preliminary results suggest SUN/NMCP interactions, a SUN link with chromatin remains to be investigated (Graumann et al., 2013). However, NMCP/LINC, SUN, and WIP functional analyses reveal their involvement in maintaining the nuclear size and shape in differentiated cells (Dittmer et al., 2007; Oda and Fukuda, 2011; Zhou et al., 2012).

As AtGIPs are found at the NE close to NPCs (Figure 5), and considering the altered NPC and AtSUN1 distribution in *gip1gip2* mutants, a structural relationship between the plant SUN1, nucleoporins and GIPs probably exists. Indeed, *gip1gip2* mutants share developmental phenotypes (Janski et al., 2012) with nucleoporin mutants, such as dwarfism, short roots and sterility (Parry, 2013), while *sun1* mutants remain fertile (Zhou et al., 2012).

Altogether our data suggest the implication of AtGIPs in nuclear shaping and size control. The pleiotropic developmental defects observed in *gip1gip2* mutants (Janski et al., 2012) may reflect deep changes in developmental programmes, probably linked to modifications in the spatial regulation of the nuclear architecture.

### AtGIPs-AtTSA1 complexes

AtTSA1 is structurally related to two proteins, At*NAI2 (At3g15950)*, an ER body component and the At3g15960 protein, predicted to be involved in DNA mismatch repair (Yamada et al., 2008). Interestingly, even though the three proteins share a highly conserved C-terminal domain (Figure S2), only AtTSA1 was identified in the conditions of our yeast two-hybrid screen. The N-terminal half of AtTSA1 is composed of 10 repeats of an EFE motif, as described by Suzuki et al. (2005). This sequence is unique to plants and has been shown to mediate AtTSA1 multimerization. Consistently, 3 out of the 10 EFE repeats are predicted to promote coiled-coil structures (Figure 6A, underlined sequences), which are frequently involved in protein-protein interactions (Rose et al., 2004; Wang et al., 2012). The C-terminal sequence of AtTSA1 also displays a coiled-coil motif, which could play a role in its interaction with AtGIPs. In our yeast two-hybrid growth assay, both AtGIPs can interact with AtTSA1 domains. However, under high stringent conditions, the largest AtTSA1 domain (D1) is only able to interact with AtGIP1 and no longer with AtGIP2 (Figure S3). This suggests that AtGIPs may be part of different subcellular complexes including AtTSA1. However, we cannot exclude the implication of other actors in promoting and/or stabilizing the AtGIP-AtTSA1 association.

AtTSA1 also possesses a putative transmembrane (TM) domain close to its C-terminal region (Suzuki et al., 2005), as well as an adjacent VIPT motif resembling the highly conserved ϕ-VPT motif found in the WIP KASH domain proteins (ϕ, hydrophobic amino acid). The ϕ-VPT motif of AtWIP1 is essential for its NE localization, and a VIPT motif is also found at the C-terminal tail of AtWIP3 located in the perinuclear space (Xu et al., 2007). It must be noted that this motif is absent from both AtNAI2 and At3g15960 proteins. Altogether, these data suggest that AtTSA1 may be addressed to and/or retained at the NE. Consistently, in plants co-expressing AtTSA1-RFP and AtGIP1-GFP, AtTSA1 was found at the NE in interphase cells with a similar punctate localization pattern as that observed for AtGIP1 (Figure 7).

### Modeling a nucleo-cytoplasmic continuum

The NE has for long solely been considered as a selective barrier between the nucleoplasm and the cytoplasm of eukaryotic cells. However, the recent discovery of evolutionarily conserved LINC complexes, spanning the double membrane and involved in essential cellular processes, confers unsuspected versatility to the NE. The ONM is continuous with the ER and shares many of its proteins. AtTSA1 harbors an N-terminal signal peptide, indicating that the protein is initially targeted to the ER (Martoglio and Dobberstein, 1998) and as shown in this study, located at the NE. According to known cotranslational translocation processes (Hegde and Kang, 2008), AtTSA1 may be integrated in the ONM through its transmembrane domain, with its C-terminal part protruding on the cytoplasmic side. An AtGIP-AtTSA1 interaction may then be involved in the association of the γ-TuCs to the outer NE. The above described ϕ-VPT motif found in AtWIP1 is furthermore required for its interaction with the AtSUNs (Zhou et al., 2012; Tamura et al., 2013). Similarly, the VIPT motif of AtTSA1 may be important for its NE localization and retention, through a possible interaction with AtSUN1 and/or AtSUN2 proteins in the perinuclear space.

The AtTSA1 interacting partner TSK, also named BRU1/MGO3 in subsequent studies, is an epigenetic nuclear factor which plays an important role in genome and chromatin maintenance (Suzuki et al., 2004; Takeda et al., 2004; Guyomarc'h et al., 2006; Ohno et al., 2011). The AtTSK interaction with AtTSA1 in interphase would imply that AtTSA1 could also be located at the INM. As the domains of AtTSA1 interacting with AtTSK and AtGIPs are partly overlapping, there is the possibility that AtTSA1, AtTSK, and/or AtGIPs may be part of specific protein complexes, located close to the INM and involved in chromatin regulation. This is in agreement with the observed nuclear localization of a fraction of AtGIPs in interphase nuclei (Janski et al., 2012).

More recently, AtTSA1 was also described to interact with the N-terminal domain of CSN1, a subunit of the COP9 signalosome in Arabidopsis (Li et al., 2011). The CSN1-NTD is specifically required for the nuclear localization of the E3-ubiquitin ligase COP1 (Wang et al., 2009) which plays a major role in controlling light-induced chromatin decondensation (van Zanten et al., 2012). This is an additional indication for a role of AtTSA1 within the nuclear compartment.

Altogether, this study provides a first insight into the role of AtGIPs in determining the NE morphology. Our findings, in addition to previous results (Janski et al., 2012; Nakamura et al., 2012), indicate that these small proteins may have a dual function, both as components of MT nucleation complexes and also as adaptors and/or modulators of NE associated proteins. Elucidating AtGIP functions may help to decipher the complex molecular interplay established at the nucleo-cytoplasmic interface.

## Author contributions

Morgane Batzenschlager and Kinda Masoud carried out the main research stated in the paper, obtained the results and analyzed the data; Natacha Janski first characterized the GIP interactors; Marie-Edith Chabouté proposed the concept of GIP's role in a nucleo-cytoplasmic continuum and developed the tools used in this work; Etienne Herzog and Marie-Edith Chabouté supervised the obtaining of data and analyzed the results with the PhD students; Yves Nominé and Bruno Kieffer performed the physical approach (CD); Jean-Luc Evrard performed the sequence comparisons and computer modeling; Guy Houlné contributed to the part of establishing transgenic lines; Mathieu Erhardt carried out the electron microscopy approach; Nicolas Baumberger performed the biochemical approaches; Anne-Catherine Schmit performed and supervised the microscopy approaches; Marie-Edith Chabouté, Anne-Catherine Schmit, Morgane Batzenschlager, and Etienne Herzog wrote the paper.

## Conflict of interest statement

The authors declare that the research was conducted in the absence of any commercial or financial relationships that could be construed as a potential conflict of interest.

## Supplementary material

Figure S1 **Detail of the distribution of AtGIP1-GFP, chromatin, EYFP-AtCENH3 and microtubules in an Arabidopsis root cell. (A)** Fluorescent image after α-tubulin immunolabeling (red) and DAPI staining (blue) in a cell expressing AtGIP1-GFP (green). **(B)** Corresponding drawing showing a perinuclear AtGIP1-GFP dot at a MT minus end close to chromocenters. The yellow square points out the colocalization between the minus end of a percinuclear MT and GIP1-GFP. Bar = 1.5 μm. **(C–J)** Fluorescent images after α-tubulin immunolabeling (red), and DAPI staining (blue) in cells expressing EYFP-CENH3 (green) arrows in **(C–E)**. **(H,K)** Corresponding drawings showing MT minus ends close to EYFP-CENH3 signals and chromocenters.

Figure S2 **Multiple sequence alignment of AtTSA1 and its two homologs in Arabidopsis**. The sequences were aligned using the ClustalW multiple alignment tool. (*) identical amino acids, (.) similar amino acids. The numbers show the amino acid residue positions. Both the signal peptide (aa 1–29) and the C-terminal region (aa 513–755) of AtTSA1 show sequence homology with AtNAI2 and At3g15960 proteins. Large gaps in the alignment are due to the lack of EFE repeats (aa 91–469) in At3g15960 protein.

Figure S3 **Comparative analysis of AtGIP1 and AtGIP2 interaction with AtTSA1 domains in a yeast two-hybrid growth assay**. Full-length AtGIP1 and AtGIP2 are fused to the Gal4 DNA Binding domain (BD, bait) while the longest (TSA1-D1) and minimal (TSA1-D4) domains of AtTSA1 identified by yeast two-hybrid screening are fused to the Gal4 Activation domain (AD, prey). AH109 cells co-transformed with the different recombinant vectors were spotted directly and after ten-fold serial dilutions onto control (-LW) and selection media (− HLW + 3-AminoTriazole, 3-AT and -AHTL) of increasing stringency. Growth results are shown after a 2-day incubation at 30°C and were obtained for 4 individual colonies per combination. The interaction of AtGIP1 with both D1 and D4 domains of AtTSA1 is confirmed by the growth of cells even on high-stringency (-AHTL) media. On the other hand, a slower growth of AH109 transformants carrying AtGIP2 and TSA1-D1 recombinant proteins compared to the AtGIP2/TSA1-D4 combination indicates a differential requirement for AtTSA1 domains to establish the interaction between AtTSA1 and AtGIP proteins.

Figure S4 **AtGIP1 interacts with itself and AtGIP2 in a yeast two-hybrid system**. Yeast strain Y187 was co-transformed with plasmids encoding AtGIP1 fused to the Gal4 Activation domain (AD), and either AtGIP1 or AtGIP2 fused to the Gal4 DNA Binding domain (BD). Protein interactions were assessed in a β-galactosidase filter assay (Janski et al., 2008), with the appearance of a blue color indicating interaction between the proteins tested. The retinoblastoma protein (Rb1) of maize (*Zea mays*) and the RepA protein of wheat (*Triticum aestivum*) dwarf geminivirus (geminivirus of the genus *Mastrevirus* which infects monocotyledonous plants) were used as positive controls (Xie et al., 1996). Yeasts co-transformed with AtGIP recombinant plasmids and empty AD or BD two-hybrid vectors were used as negative controls. Both AtGIP1-AtGIP1 and AtGIP1-AtGIP2 interactions were detected.

Table S1 **List of primers used in cloning experiments**.

Table S2 **Accession numbers of the sequences used for establishing the phylogenetic tree presented in Figure 1**. Note: The previous study predicted fission yeast MOZART1 homolog MZT1 to be a 97 amino acid protein (Hutchins et al., 2010; Dhani et al., 2013; Masuda et al., 2013), have shown that the initiator methionine corresponds to the methionine 34 of the previously annotated MZT1 ORF. Consequently, MZT1 is a protein of 64 amino acids similar to MOZART1 homologs from other species.
