# Supplementary material for: The GIP gamma-tubulin complex-associated proteins are involved in nuclear architecture in Arabidopsis thaliana
Source: Front Plant Sci. 2013 Nov 27;4:480. doi: 10.3389/fpls.2013.00480 (PMC3842039; doi:10.3389/fpls.2013.00480)
Supplement: Figure S1 — Detail of the distribution of AtGIP1-GFP, chromatin, EYFP-AtCENH3 and microtubules in an Arabidopsis root cell. (A) Fluorescent image after α-tubulin immunolabeling (red) and DAPI staining (blue) in a cell expressing AtGIP1-GFP (green). (B) Corresponding drawing showing a perinuclear AtGIP1-GFP dot at a MT minus end close to chromocenters. The yellow square points out the colocalization between the minus end of a percinuclear MT and GIP1-GFP. Bar = 1.5 μm. (C–J) Fluorescent images after α-tubulin immunolabeling (red), and DAPI staining (blue) in cells expressing EYFP-CENH3 (green) arrows in (C–E). (H,K) Corresponding drawings showing MT minus ends close to EYFP-CENH3 signals and chromocenters. [file DataSheet1.ZIP › Schmit/Supplementary Table 1.pdf]

Supplementary Table S1

Vector	Forward primer (F-) Reverse primer (R-)
Cloning of the *GIP1* (At4g09550) cDNA	
pAS2ΔΔ	F- CATGCCATGGCATGTATGGATGAGGAGGCATCTCGG<br>R- CGCGGATCCGCGTCAGTGTATAGATGGTGTGGTTGTGAC
pET102D	F- CACCATGGATGAGGAGGCATC<br>R- TCAGTGTATAGATGGTG
PCR-based mutagenesis	
pET102D-GIP1	F- GCAAGAGAGTCACTAGAG<br>R- ATGGTGATGGTGATGATGTCCCATTATGTATATCTC
Cloning of the *GIP1* (At4g09550) promoter and cDNA	
pDONR207	F- GGGGACAAGTTTGTACAAAAAAGCAGGCTTCCTCCTACACCGAAATAG 3<br>R- GGGGACCACTTTGTACAAGAAAGCTGGGTTGTGTATAGATGGTGTGG
Cloning of the *GIP2* (At1g73790) promoter and cDNA	
pDONR207	F- GGGGACAAGTTTGTACAAAAAAGCAGGCTTCGCCAGTACTTGAAGAACC<br>R- GGGGACCACTTTGTACAAGAAAGCTGGGTTATCAACCGTAGTTGTTGTTG
