# Supplementary material for: The GIP gamma-tubulin complex-associated proteins are involved in nuclear architecture in Arabidopsis thaliana
Source: Front Plant Sci. 2013 Nov 27;4:480. doi: 10.3389/fpls.2013.00480 (PMC3842039; doi:10.3389/fpls.2013.00480)
Supplement: Figure S1 — Detail of the distribution of AtGIP1-GFP, chromatin, EYFP-AtCENH3 and microtubules in an Arabidopsis root cell. (A) Fluorescent image after α-tubulin immunolabeling (red) and DAPI staining (blue) in a cell expressing AtGIP1-GFP (green). (B) Corresponding drawing showing a perinuclear AtGIP1-GFP dot at a MT minus end close to chromocenters. The yellow square points out the colocalization between the minus end of a percinuclear MT and GIP1-GFP. Bar = 1.5 μm. (C–J) Fluorescent images after α-tubulin immunolabeling (red), and DAPI staining (blue) in cells expressing EYFP-CENH3 (green) arrows in (C–E). (H,K) Corresponding drawings showing MT minus ends close to EYFP-CENH3 signals and chromocenters. [file DataSheet1.ZIP › Schmit/Supplementary Table 2.pdf]

Supplementary Table S2

Scientific Name	Ref. Database	size (aa)	Scientific Name	Ref. Database	size (aa)
Arabidopsis thaliana	At4g09550.1	71	*Bos taurus*	gb|XP_872775.1	80
Arabidopsis thaliana	At1g73790	67	*Branchiostoma floridae*	gb|EEA55013.1	71
Ostreococcus lucimarinus	gb|ABO99814.1	61	*Ciona intestinalis*	gb|FF977949.1	65
Ostreococcus tauri	emb|CAL57683.1	61	*Cryptosporidium hominis*	gb|EAL37150.1	66
Physcomitrella patens	dbj|DC931782.1	68	*Cryptosporidium muris*	gb|EEA06029.1	67
Physcomitrella patens	gb|FC379761.1	68	*Drosophila melanogaster*	gb|HDC08084.1	82
Picea sitchensis	gb|DR534840.1	69	*Drosophila sechellia*	gb|DK308718.1	82
Populus trichocarpa	gb|CV246545.1	74	*Drosophila willistoni*	gb|GK12642.1	79
Vitis vinifera	gb|EC991719.1	73	*Equus caballus*	ref|XP_001489819.1	82
Aspergillus clavatus	gb|EAW07904.1	71	*Gallus gallus*	ref|NP_001157822.1	72
Aspergillus nidulans	gb|EAA65544.1	74	*Homo sapiens*	gb|EAW80513.1	79
Botryotinia fuckeliana	gb|EDN28486.1	83	*Macaca fascicularis*	gb|BAE90266.1	82
Chaetomium globosum	gb|EAQ86685.1	87	*Monodelphis domestica*	ref|XP_001362297.1	82
Coccidioides immitis	gb|EER28139.1	72	*Mus musculus*	gb|CJ136191.1	78
Coprinopsis cinera	gb|EAU90559.1	75	*Ornithorhynchus anaticus*	ref|XP_001514347.1	79
Cryptococcus neoformans	gb|EAL23521.1	72	*Paramecium tetraurelia*	emb|CAK87619.1	85
Gibberella zeae	gb|CN813262.1	73	*Plasmodium berghei*	emb|CAI01583.1	76
Laccaria bicolor	gb|EDR14655.1	70	*Plasmodium falciparum*	emb|CAD52532.1	72
Magnaporthe grisea	gb|EDJ97074.1	80	*Plasmodium knowlesi*	emb|CAQ41220.1	76
Moniliophthora perniciosa	gb|EEB91345.1	71	*Plasmodium vivax*	gb|EDL44454.1	76
Neosartorya Fischeri	gb|EAW22942.1	77	*Rattus norvegicus*	ref|XP_001081273.1	78
Penicillium chrysogenum	emb|CAP92908.1	77	*Taeniopygia guttata*	gb|FE727619.1	72
Penicillium marneffei	gb|EEA27701.1	70	*Tetraodon nigroviridis*	emb|CAG10085.1	66
Phaeosphaeria nodorum	gb|EAT83926.1	82	*Theileria annulata*	emb|CAI75874.1	84
Pyrenophora tritici-repentis	gb|EDU42052.1	82	*Toxoplasma gondii*	gb|EEA98898.1	92
Sclerotinia sclerotiorum	gb|EDN94334.1	83	*Trichomonas vaginalis*	gb|EAX84752.1	67
Saccharomices pombe	gb|429242268	97	*Xenopus laevis*	gb|BG162997.1	72
Babesia bovis	gb|EDO07752.1	91	*Xenopus tropicalis*	gb|CN103772.1	72
