# Supplementary figures and images for: The GIP gamma-tubulin complex-associated proteins are involved in nuclear architecture in Arabidopsis thaliana
Source: Front Plant Sci. 2013 Nov 27;4:480. doi: 10.3389/fpls.2013.00480 (PMC3842039; doi:10.3389/fpls.2013.00480)

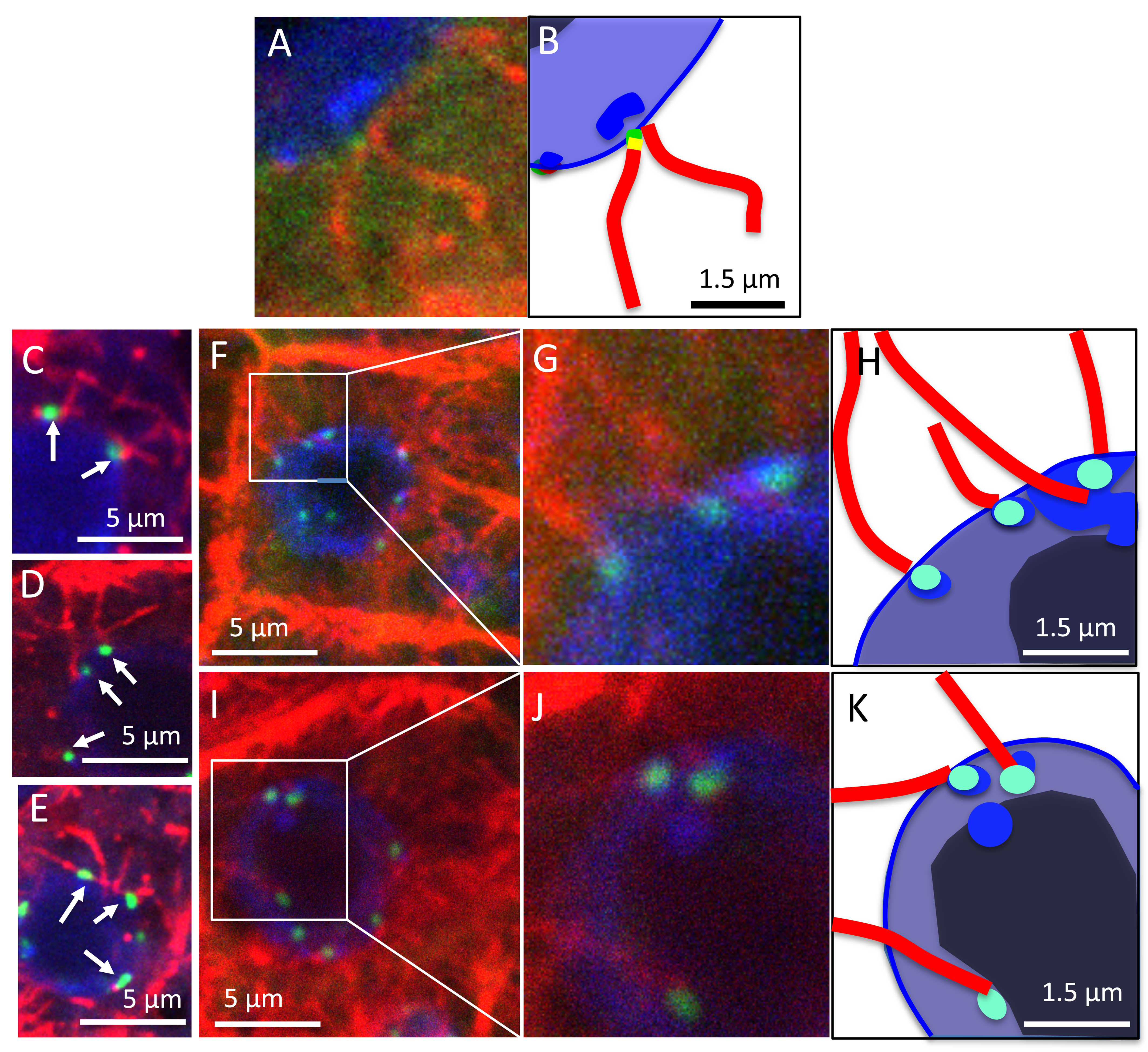

Supplement: Figure S1 — Detail of the distribution of AtGIP1-GFP, chromatin, EYFP-AtCENH3 and microtubules in an Arabidopsis root cell. (A) Fluorescent image after α-tubulin immunolabeling (red) and DAPI staining (blue) in a cell expressing AtGIP1-GFP (green). (B) Corresponding drawing showing a perinuclear AtGIP1-GFP dot at a MT minus end close to chromocenters. The yellow square points out the colocalization between the minus end of a percinuclear MT and GIP1-GFP. Bar = 1.5 μm. (C–J) Fluorescent images after α-tubulin immunolabeling (red), and DAPI staining (blue) in cells expressing EYFP-CENH3 (green) arrows in (C–E). (H,K) Corresponding drawings showing MT minus ends close to EYFP-CENH3 signals and chromocenters. [file DataSheet1.ZIP › Schmit/70531_Schmit_Figure_S1.TIF]

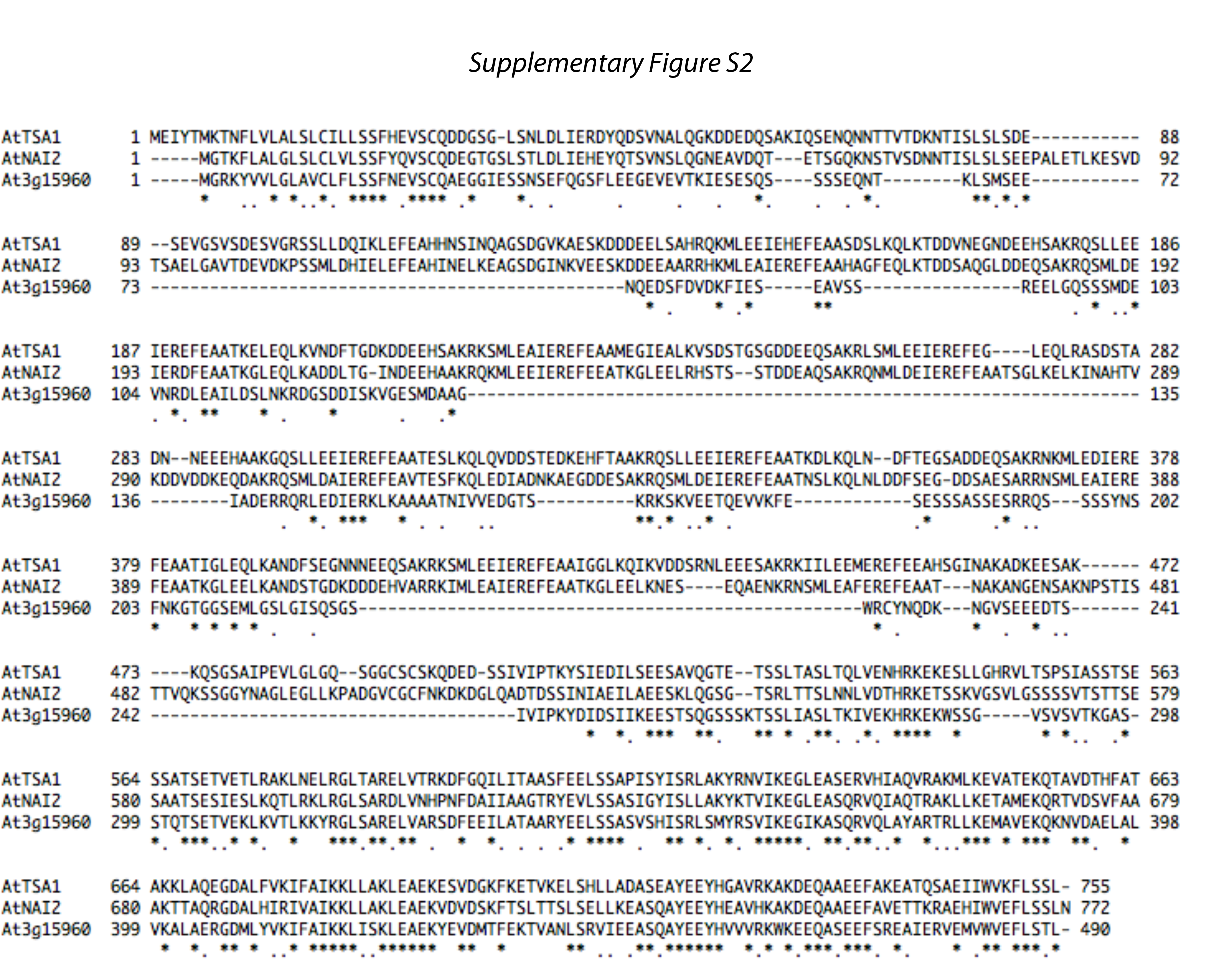

Supplement: Figure S1 — Detail of the distribution of AtGIP1-GFP, chromatin, EYFP-AtCENH3 and microtubules in an Arabidopsis root cell. (A) Fluorescent image after α-tubulin immunolabeling (red) and DAPI staining (blue) in a cell expressing AtGIP1-GFP (green). (B) Corresponding drawing showing a perinuclear AtGIP1-GFP dot at a MT minus end close to chromocenters. The yellow square points out the colocalization between the minus end of a percinuclear MT and GIP1-GFP. Bar = 1.5 μm. (C–J) Fluorescent images after α-tubulin immunolabeling (red), and DAPI staining (blue) in cells expressing EYFP-CENH3 (green) arrows in (C–E). (H,K) Corresponding drawings showing MT minus ends close to EYFP-CENH3 signals and chromocenters. [file DataSheet1.ZIP › Schmit/70531_Schmit_Figure_S2.TIF]

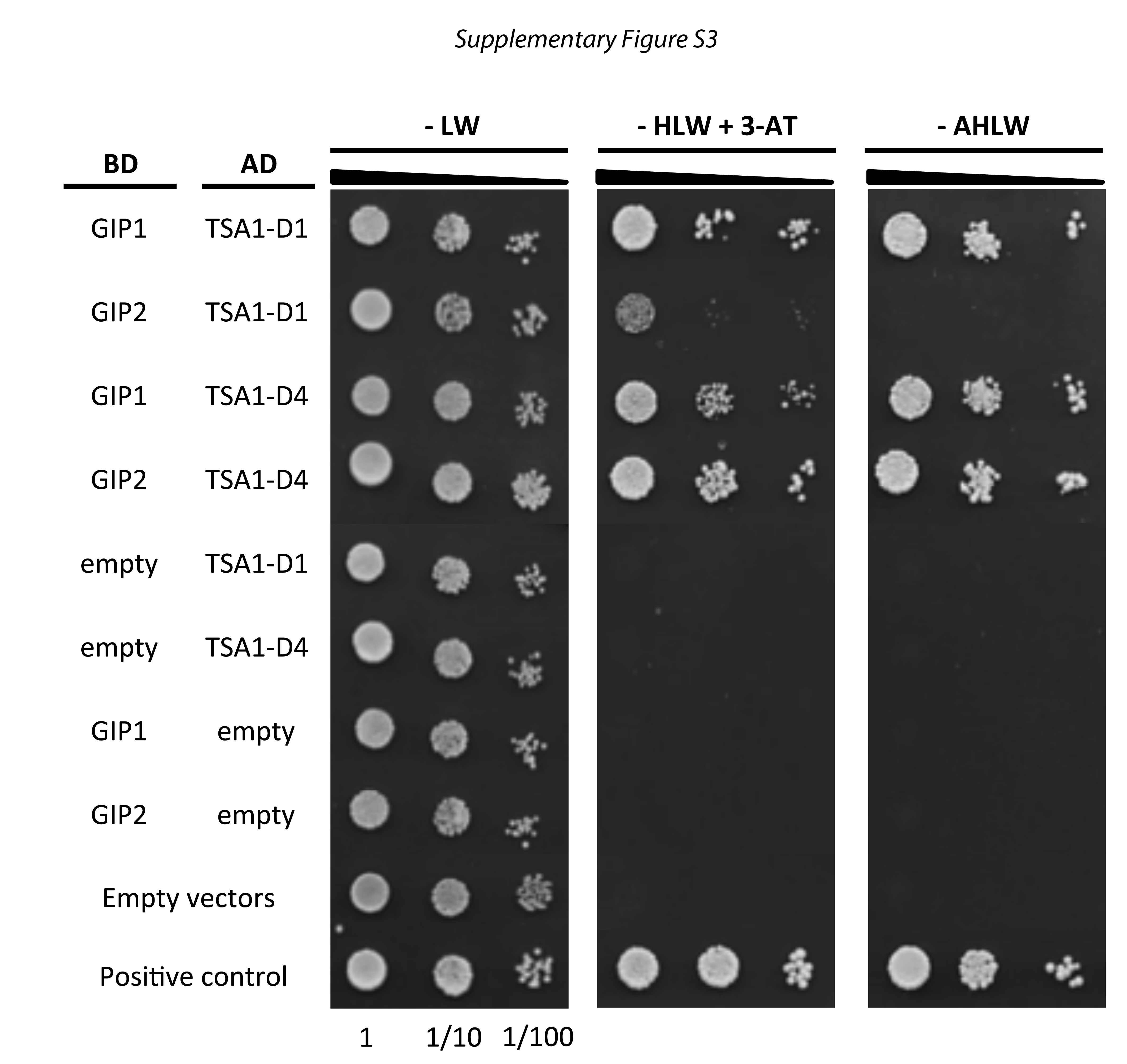

Supplement: Figure S1 — Detail of the distribution of AtGIP1-GFP, chromatin, EYFP-AtCENH3 and microtubules in an Arabidopsis root cell. (A) Fluorescent image after α-tubulin immunolabeling (red) and DAPI staining (blue) in a cell expressing AtGIP1-GFP (green). (B) Corresponding drawing showing a perinuclear AtGIP1-GFP dot at a MT minus end close to chromocenters. The yellow square points out the colocalization between the minus end of a percinuclear MT and GIP1-GFP. Bar = 1.5 μm. (C–J) Fluorescent images after α-tubulin immunolabeling (red), and DAPI staining (blue) in cells expressing EYFP-CENH3 (green) arrows in (C–E). (H,K) Corresponding drawings showing MT minus ends close to EYFP-CENH3 signals and chromocenters. [file DataSheet1.ZIP › Schmit/70531_Schmit_Figure_S3.TIF]

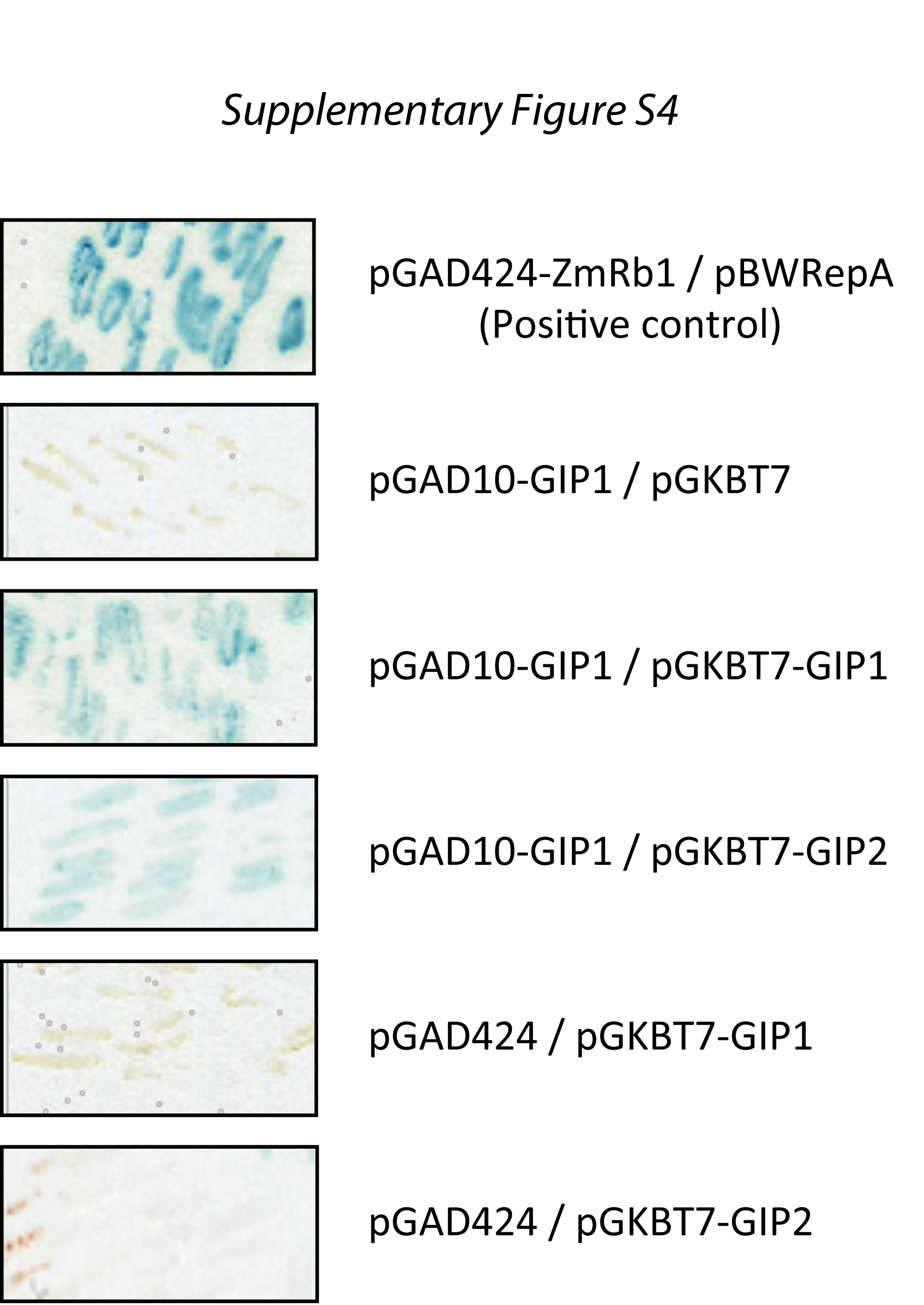

Supplement: Figure S1 — Detail of the distribution of AtGIP1-GFP, chromatin, EYFP-AtCENH3 and microtubules in an Arabidopsis root cell. (A) Fluorescent image after α-tubulin immunolabeling (red) and DAPI staining (blue) in a cell expressing AtGIP1-GFP (green). (B) Corresponding drawing showing a perinuclear AtGIP1-GFP dot at a MT minus end close to chromocenters. The yellow square points out the colocalization between the minus end of a percinuclear MT and GIP1-GFP. Bar = 1.5 μm. (C–J) Fluorescent images after α-tubulin immunolabeling (red), and DAPI staining (blue) in cells expressing EYFP-CENH3 (green) arrows in (C–E). (H,K) Corresponding drawings showing MT minus ends close to EYFP-CENH3 signals and chromocenters. [file DataSheet1.ZIP › Schmit/70531_Schmit_Figure_S4.TIF]
